# Reassessing CAC Risk Post-RRSO: The Overlooked Role of Hormone Therapy

**DOI:** 10.1016/j.jaccao.2024.12.008

**Published:** 2025-03-18

**Authors:** Binglin Li, Yan Wang, Ruijuan Chen

**Affiliations:** Xi’an Central Hospital, Xi’an, Shaanxi, China

We commend Beekman et al^1^ for their thorough exploration of coronary artery calcium (CAC) scores in women after premenopausal and postmenopausal risk-reducing salpingo-oophorectomy (RRSO). Their finding that premenopausal RRSO does not adversely affect long-term CAC scores provides reassurance for patients and clinicians. However, we believe there are areas warranting further investigation to fully understand these findings.

First, the conclusion that surgical menopause does not elevate CAC risks might overlook the role of menopausal hormone therapy. The investigators noted that menopausal hormone therapy use was higher in the premenopausal group but did not significantly influence CAC outcomes.^1^ However, prior studies suggested that the timing, dose, and type of menopausal hormone therapy critically modify cardiovascular outcomes.^2^ A more granular analysis stratifying menopausal hormone therapy users by these factors might yield further insights into its protective or neutral effects on CAC development.

Second, the investigators excluded participants with prior percutaneous coronary interventions or mechanical valves but included those with breast cancer and radiotherapy. Notably, internal mammary chain radiotherapy is a known risk factor for ischemic heart disease.^3^ Stratifying by radiotherapy status or excluding these patients could help clarify whether RRSO independently influences CAC scores or if the findings were confounded by cancer treatment.

Last, the investigators emphasize the potential differences between surgical and natural menopause on cardiovascular risk. This distinction raises important questions about the biological mechanisms underlying accelerated vascular aging in natural menopause compared with its absence in surgical menopause. Investigating markers of endothelial function or inflammatory cytokines might elucidate why these 2 processes diverge, aligning with findings in other high-risk populations.^4^ Estrogen’s well-documented protective effects on endothelial function and cardiovascular health, as described by Mendelsohn and Karas^5^ in 1999, could provide insights into why early natural menopause correlates with increased cardiovascular risks, whereas early surgical menopause appears less detrimental in this context.

By addressing these considerations, future studies could refine risk assessments and enhance individualized care for women undergoing RRSO.
